# A Comprehensive Evaluation of Patient Satisfaction and Clinical Outcomes in Poly‐L‐Lactic Acid Treatments—A Randomized, Interventional, and Comparative Clinical Study

**DOI:** 10.1111/jocd.70891

**Published:** 2026-05-05

**Authors:** Victor R. M. Munoz‐Lora, Danielle Dias, Fernanda Bezerra, Ana C. N. Carnevali, Rud Varela, Andrea D. Tedesco, Pietra Roschel, Gabriela Giro, Patrícia Pauletto, Victor Rogerio, Marcelo Germani

**Affiliations:** ^1^ Department of Facial Aesthetics Guarulhos University Guarulhos São Paulo Brazil; ^2^ HOF Pro Academy Rio Verde Goiás Brazil; ^3^ University of Brasília Brasília Brazil; ^4^ Private Office Brasília Brazil; ^5^ US Orofacial Clinic Brasília Brazil; ^6^ Department of Endocrinology and Metabolism Federal University of São Paulo São Paulo Brazil; ^7^ Private Office São Paulo Brazil; ^8^ IAT Instituto Andrea Tedesco Rio de Janeiro Brazil; ^9^ Universidad de Las Américas Quito Ecuador; ^10^ Department of Biological Sciences Bauru School of Dentistry University of São Paulo Bauru São Paulo Brazil

**Keywords:** facial aesthetics, facial anatomy, poli‐L‐lactic‐acid, ultrasound

## Abstract

**Background:**

Poly‐L‐lactic acid (PLLA) is widely used in aesthetic medicine due to its bio‐stimulatory properties. However, limited research has compared different PLLA formulations.

**Aim:**

To evaluate the impact of PLLA formulation and injection depth (subcutaneous vs. supraperiosteal) using objective and subjective assessments.

**Materials and Methods:**

This randomized clinical trial with split‐face and parallel components included 20 participants. Each participant received a single PLLA formulation of PLLA‐SCA (Sculptra) or PLLA‐ELL (Elleva), while the injection plane was alternated between hemifaces. Dermal thickness was assessed using ultrasonography at baseline, 60, and 120 days. Patient satisfaction was evaluated using the Global Aesthetic Improvement Scale (GAIS).

**Results:**

Dermal thickness increased over time but showed no significant differences between products (*p* = 0.78) or injection planes (*p* = 0.83). GAIS scores were positive at 60 days but declined significantly at 120 days (*p* = 0.031), particularly for PLLA‐ELL (*p* = 0.022). No significant correlation was found between dermal thickness and GAIS scores at 60 (*ρ* = −0.038, *p* = 0.819) or 120 days (*ρ* = 0.016, *p* = 0.927).

**Conclusions:**

Injection depth did not significantly influence PLLA outcomes. However, differences in patient satisfaction between PLLA formulations highlight the role of physicochemical properties in treatment longevity. These findings emphasized the need to integrate objective and subjective measures in assessing bio‐stimulatory treatments.

## 1 Introduction

Patient satisfaction is a critical outcome in facial aesthetic treatments, as it depends on the ability to perceive subtle yet clinically meaningful improvements resulting from the procedure [1]. Currently, treatment efficacy can be measured both qualitatively and quantitatively [2]. In most cases, qualitative analyses are performed by both patients and evaluators, while quantitative assessments are conducted exclusively by trained operators. The introduction of highly sensitive equipment, such as high‐frequency ultrasonography, has allowed for the detection of subtle dermal changes, initially attracting the attention of clinicians and researchers aiming to enhance evaluation objectivity [3]. However, the exclusive reliance on quantitative methods, while eliminating subjectivity, may carry the risk of misinterpreting clinical outcomes, as these approaches often fail to capture the patient's perception of treatment success [4, 5].

Poly‐L‐lactic acid (PLLA) is a well‐established bio‐stimulator used in facial aesthetic treatments due to its ability to stimulate collagen production and improve skin quality, elasticity, and hydration [6]. Despite its widespread use, there is still limited research directly comparing different PLLA formulations, their clinical effects, and how they influence patient satisfaction. Additionally, the impact of the injection plane (subcutaneous vs. supraperiosteal) on treatment outcomes remains unclear. While previous studies have confirmed the bio‐stimulatory effects of PLLA, a recent systematic review has highlighted concerns regarding the low quality of evidence supporting its long‐term efficacy and safety [7, 8]. These findings underscore the need for further clinical investigations utilizing both objective and subjective assessments to comprehensively evaluate treatment effectiveness.

To address this gap, this study aimed to compare two different PLLA formulations and investigate the impact of injection depth (subcutaneous vs. supraperiosteal) on treatment outcomes. Evaluations were performed using high‐frequency ultrasonography to measure dermal thickness and the Global Aesthetic Improvement Scale (GAIS) to assess patient‐perceived outcomes. By correlating objective imaging findings with subjective patient satisfaction scores, this study seeks to determine how different formulations and application techniques influence the aesthetic outcomes of PLLA treatments.

## 2 Materials and Methods

### 2.1 Participants

Twenty female participants seeking facial aesthetic treatments using biostimulators were recruited from a private clinic in the city of Lins, São Paulo, Brazil, between January 2024 and May 2024. The sample size was determined based on feasibility and on sample sizes commonly reported in comparable imaging‐based PLLA studies [7, 9]. Participants with preexisting medical conditions, such as autoimmune diseases, severe neuromuscular disorders, active or chronic inflammatory skin conditions, a history of keloid formation, blood coagulation disorders, known allergies to the product components, active infections in the proposed treatment area, pregnancy or breastfeeding, and those who had undergone any type of aesthetic treatment involving biostimulators, fillers, or other regenerative procedures in the past 12 months were excluded from the study. Patients who had undergone facial surgeries within the last 2 years or were using chronic corticosteroid or immunosuppressive therapy were also excluded. Additionally, individuals who did not agree to sign an individual informed consent form before involvement were not included in the study.

All procedures followed the ethical standards of the national research committees, the Declaration of Helsinki and its subsequent amendments, or comparable ethical standards. Ethical approval for this study (CAAE: 68933023.1.0000.0191) was obtained.

### 2.2 Experimental Design

The present investigation is a randomized, interventional, controlled clinical trial. The study was reported in accordance with the Consolidated Standards of Reporting Trials (CONSORT) guidelines.

All participants were previously instructed to wash their faces with water and soap, and facial antisepsis with 2% alcoholic chlorhexidine (Riohex, Rioquímica, São Paulo, Brazil) was performed. Participants were treated with PLLA using two commercially available formulations. Product allocation was performed in a parallel‐group manner, and participants were randomly assigned to one of two groups (*n* = 10) using Random Allocation Software version 2.0 (Mahmood Saghaei), with allocation concealment maintained until the time of product preparation. Injection plane (subcutaneous vs. supraperiosteal) was evaluated using a split‐face approach, with different techniques applied to each hemiface according to the study protocol. Ultrasound assessments were performed by a single experienced operator who was blinded to the PLLA formulation used, and participants were also blinded to the product administered.

First, patients were divided into two different groups (*n* = 10) using Random Allocation 2.0 software (Mahmood Saghaei):

G1: Participants who received treatment with Sculptra (PLLA‐SCA; Galderma, Lausanne, Switzerland).

G2: Participants who received Elleva (PLLA‐ELL; Innovapharma, Goiânia, Brazil).

### 2.3 Product Preparation and Administration

Both products were reconstituted following each manufacturer's recommendations and dose equivalence between products was defined based on the total amount of PLLA (mg) administered per hemiface:

- PLLA‐SCA contains 150 mg of PLLA and was prepared immediately before treatment. First, 5 mL of sterile water (Samtec, Ribeirão Preto, São Paulo) was added to the vial, followed by vigorous manual shaking for 1 min. Then, an additional 3 mL of the same water plus 2 mL of lidocaine without vasoconstrictor (DFL, São Paulo, Brazil) were added without further shaking.
- PLLA‐ELL, which contains 210 mg of poly‐L‐lactic acid (PLLA), was prepared 1 h prior to the procedure. To prepare, 16 mL of sterile water was added into the vial. Then, a 10‐min vigorous shaking was performed using a mechanical mixer device provided by the manufacturer. The mixture was left to rest for 1 h to ensure complete hydration. Before use, 2 mL of lidocaine without vasoconstrictor was slowly added.

After reconstitution, each group received the corresponding product as follows:

- First application (day 0): Subcutaneous injection in the left hemiface and supraperiosteal injection in the right hemiface.
- Second application (day 60): Supraperiosteal injection on the left hemiface and subcutaneous injection on the right hemiface, maintaining the same product allocation for each participant.

### 2.4 Injection Techniques

**FIGURE 1:**
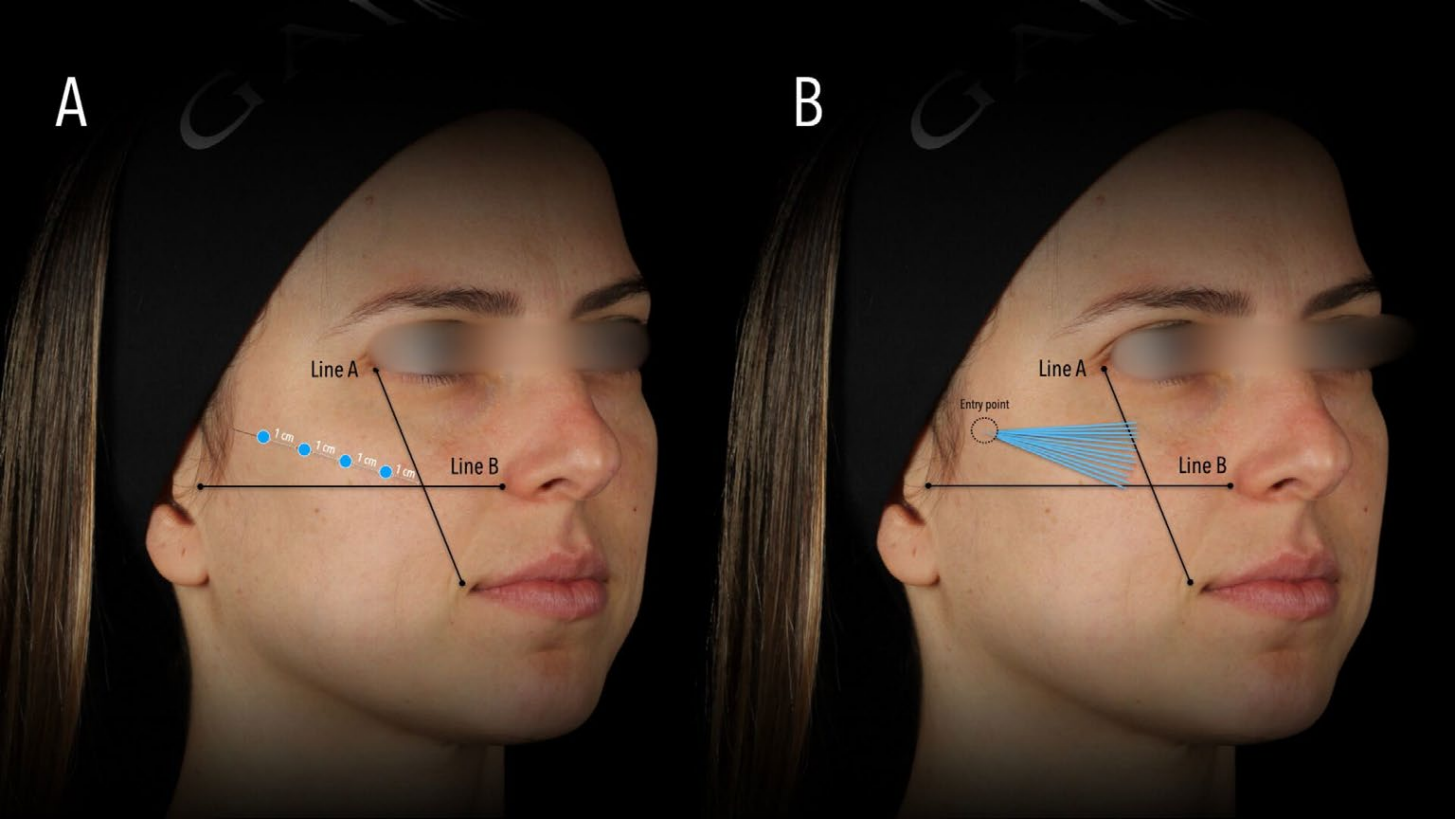
Schematic representation of poly‐L‐lactic acid (PLLA) injection techniques. (A) Supraperiosteal injection technique using a 27G needle, performed at a 90° angle until reaching the bone surface. (B) Subcutaneous injection technique using a 22G microcannula, inserted laterally and advanced medially for retroinjection in a fanning pattern.

For supraperiosteal administrations (Figure 1A), a 27G needle (Terumo, São Paulo, Brazil) adapted to the syringe containing the product was inserted into the skin at a 90° angle and advanced until it gently touched the bone surface. The first injection point was located 1 cm lateral to the intersection of the line connecting the distal corner of the eye to the labial commissure and the line connecting the tragus to the alar region of the nose. Three additional injection points were distributed laterally, maintaining a 1 cm distance among them. Each point received 0.3 mL of PLLA‐SCA (G1), corresponding to 4.5 mg of PLLA (18 mg total), or 0.4 mL of PLLA‐ELL (G2), corresponding to 4.73 mg of PLLA (18.92 mg total).

For subcutaneous administrations (Figure 1B), a 22G × 50 mm microcannula (Biometik, Santa Catarina, Brazil) was used. First, a local skin block with 2% lidocaine without vasoconstrictor (DFL, São Paulo, Brazil) was applied at the cannula entry point, located at the most lateral point used in the supraperiosteal application. The entry point was made using a 22G hypodermic needle (Terumo, São Paulo, Brazil), and the cannula was inserted subcutaneously and medially advanced. A total of six retroinjections of 0.2 mL each were performed in a fang design, representing a total of 18 mg of PLLA for PLLA‐SCA or 18.92 mg for PLLA‐ELL, ensuring nearly equal amounts of active substance.

Immediately after product administration, a gentle massage of the treated areas was performed in both groups on both sides. Participants were instructed to continue this massage at home for five consecutive days, performing five daily sessions of 5 min each.

### 2.5 Outcomes

#### 2.5.1 Dermal Thickness With Ultrasound

**FIGURE 2:**
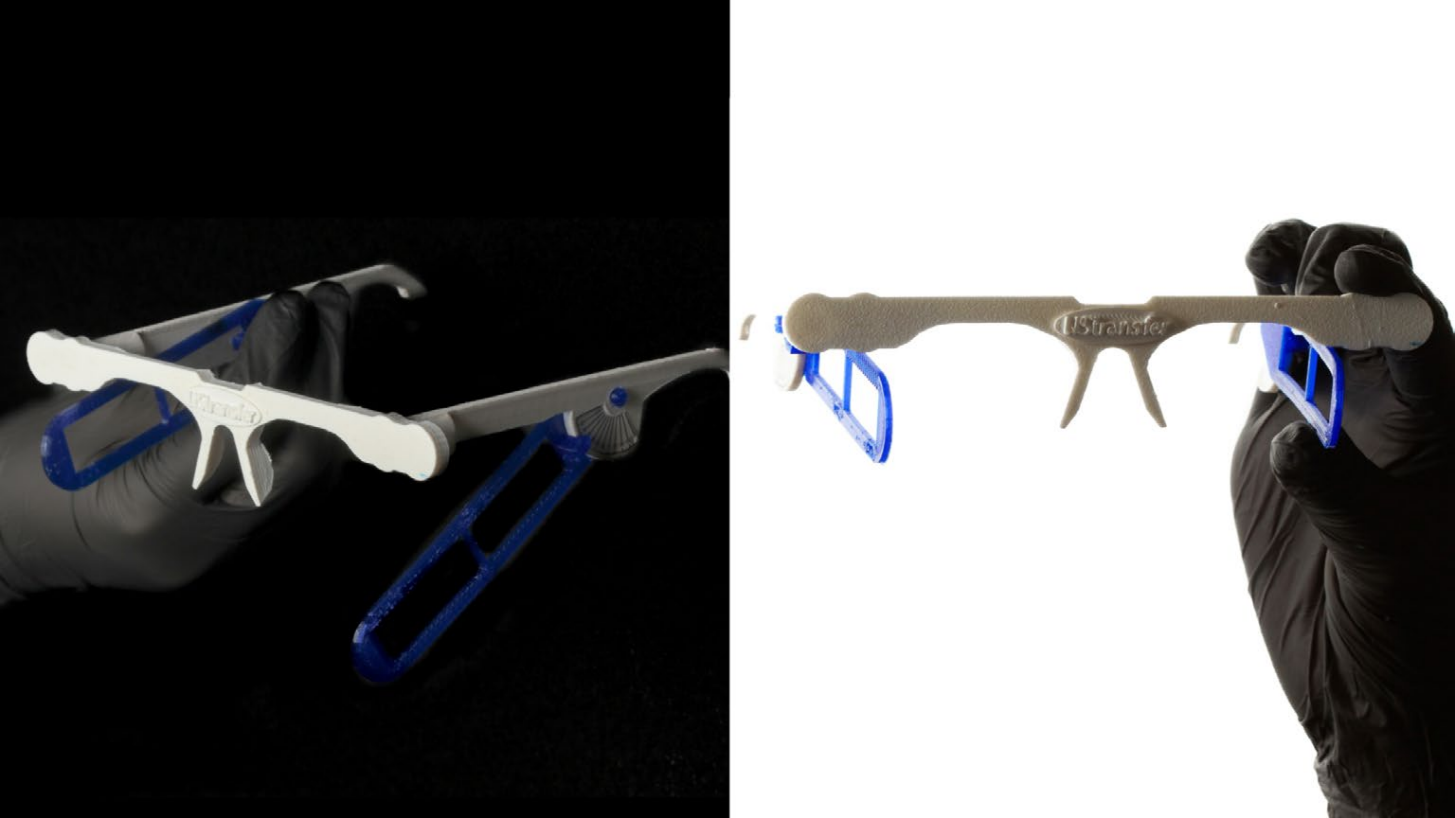
Positioning device used for standardized ultrasound acquisitions. The custom‐designed glasses‐like structure features flexible arms that ensure consistent probe placement across different time points, minimizing variability in dermal thickness measurements.

Ultrasound images were obtained from all participants before treatment (day 0), 60 days after (but before the second treatment), and 120 days after the first application (60 days after the second treatment). Dermal thickness was assessed using an ultrasound device (LOGIQ E, GE Healthcare) with 10–22 MHz and 8–18 MHz probes. The exams were performed in B‐mode, using a linear probe at a frequency of 22 MHz, positioned in the transverse/axial plane over the lateral zygomatic region. All ultrasound acquisitions were performed by a single experienced radiologist using a standardized protocol and a positioning device (UStransfer, patent: BR102022012533‐3; Figure 2) featuring a glasses‐like structure with flexible arms, facilitating accurate repetition of subsequent assessments. The examiner had extensive experience with the technique and was involved in the technical development of the positioning system, which contributed to consistent image acquisition.

#### 2.5.2 Global Assessment Improvement Scale (GAIS)

All patients were instructed to assess the obtained results using the GAIS scale, which ranges from −2 to 2, with −2 being “Much worse”, −1 “Slightly worse”, 0 “No change”, 1 “Slightly better”, and 2 “Much better”. GAIS assessments were conducted 60 and 120 days after the first treatment to evaluate the personal perception of results.

### 2.6 Statistical Analysis

All statistical analyses were conducted using Jamovi statistical software (The Jamovi Project, version 2.3.28, Sydney, Australia), with a significance level set at *p* < 0.05. All data are presented as mean ± standard deviation (SD) independent of the data format to increase readability and understandability of the results presented. All statistical tests were two‐tailed, and results were interpreted in conjunction with descriptive statistics to provide a comprehensive evaluation of treatment effects. Statistical analyses were based on pre‐specified hypotheses. Given the exploratory nature of the study and the limited number of planned comparisons, no formal correction for multiple comparisons was applied to avoid excessive conservatism in the sample.

A two‐way repeated measures ANOVA was performed to assess changes in dermal thickness over time and between products, with time (baseline, 60, and 120 days) and product (PLLA‐SCA vs. PLLA‐ELL) as factors. When necessary, post hoc comparisons were adjusted using the Tukey correction.

To evaluate GAIS, comparisons between 60 and 120 days within each treatment group were conducted using the Wilcoxon Signed Rank test, due to its non‐normal distribution. Differences in GAIS between products at each time point were assessed using the Mann–Whitney U test. Additionally, GAIS and dermal thickness comparisons between application planes (subcutaneous vs. supraperiosteal) at 60 days were evaluated using the Wilcoxon Signed Rank test.

To explore the relationship between changes in dermal thickness and patient satisfaction, Spearman's rank correlation coefficient was calculated to evaluate the association between percentage change in dermal thickness (baseline to 60 days and baseline to 120 days) and GAIS scores at 60 and 120 days. This non‐parametric correlation analysis was chosen due to the non‐normal distribution of GAIS scores.

## 3 Results

### 3.1 Demographics

The study included 20 female participants with a mean age of 44.8 ± 6.0 years [range: 38–55] and a body mass index (BMI) of 25.92 ± 2.86 kg/m^2^. G1 presented a mean age of 43.3 ± 6.49 years, while G2 had a mean age of 46.3 ± 5.19 years (*p* = 0.115). BMI values were 26.7 ± 3.55 kg/m^2^ for G1 and 25.1 ± 1.99 kg/m^2^ for G2 (*p* = 0.096), showing no significant differences between groups.

Regarding ultrasound measurements, dermal thickness at T0 was 1.27 ± 0.16 mm for G1 and 1.31 ± 0.16 mm for G2 (*p* = 0.430), with no statistically significant differences between the groups initially. Initial demographic data by groups are shown in Table 1.

**TABLE 1:** Demographics.

Demographics	Value
Participants	20
Age	44.8 ± 6.0
BMI	25.92 ± 2.86
**Product**	
Sculptra (PLLA—SCA)	10
Age	43.3 ± 6.49
BMI	26.7 ± 3.55
Dermal thickness	1.27 ± 0.16
Elleva (PLLA—ELL)	10
Age	46.3 ± 5.19
BMI	25.1 ± 1.99
Dermal thickness	1.31 ± 0.16

### 3.2 Dermal Thickness

Dermal thickness between application planes at 60 days resulted in no significant differences (*p* = 0.837), with 1.24 ± 0.223 for the subcutaneous application side and 1.22 ± 0.196 for the supraperiostial application side.

**FIGURE 3:**
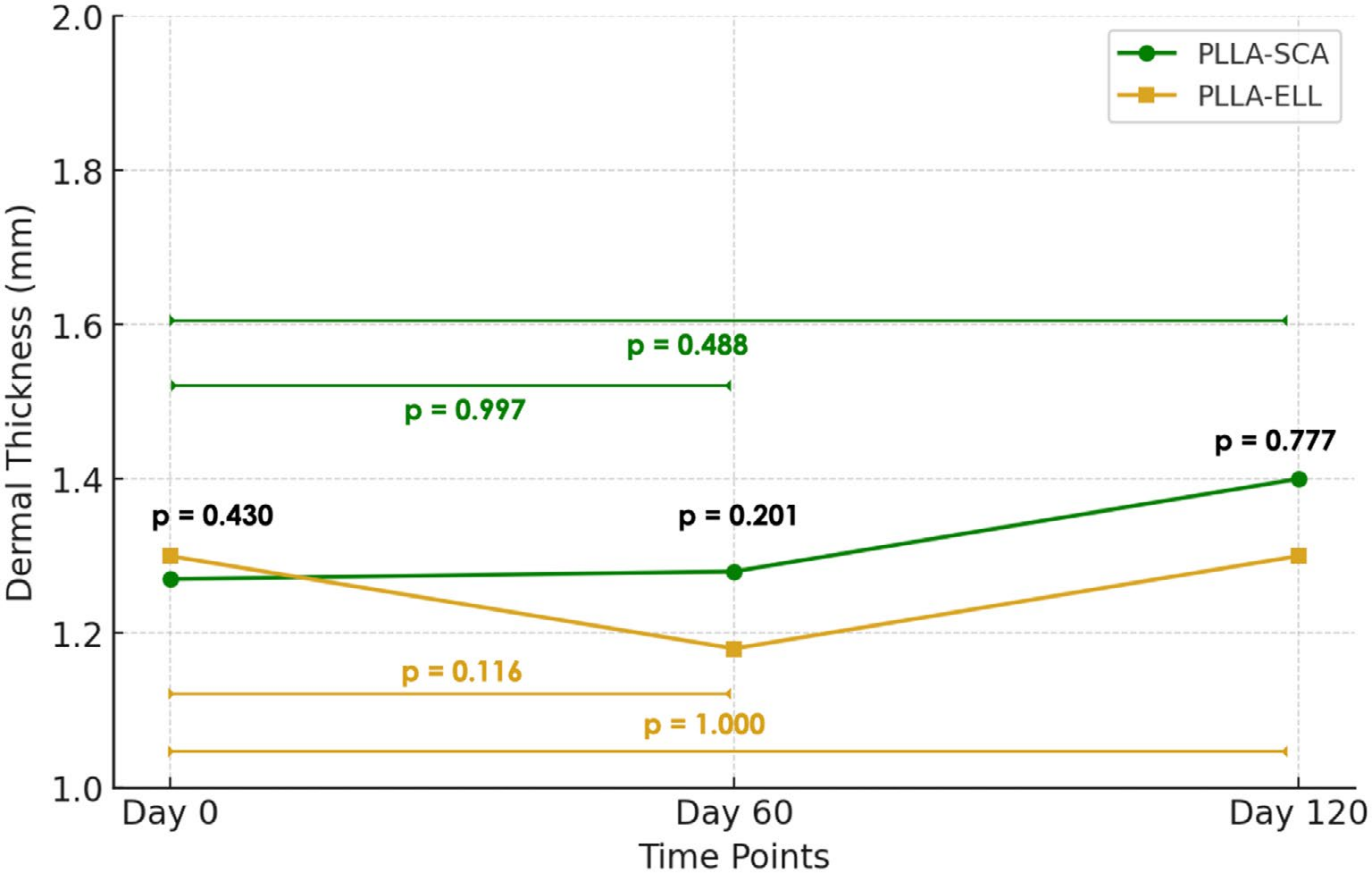
Line graph showing the mean dermal thickness (mm) of the assessed region over time (Day 0, 60, and 120), as measured by ultrasound. The data represent mean values for PLLA‐SCA and PLLA‐ELL groups. P‐values in black indicate the significance of differences between groups at each assessment time. P values in color indicate the significance of differences within groups along time.

A two‐way repeated measures ANOVA performed to examine the effect of time and product on dermal thickness resulted in no significant interactions between them (*p* = 0.161). The within‐subject effect of time indicated that even when an increment in dermal thickness was present, this was not significant (*p* = 0.063). Moreover, the between‐subject effect of product did not show a significant difference between G1 and G2 groups at 60 days (1.28 ± 0.24 for G1 and 1.18 ± 0.16 for G2; *p* = 0.20) or 120 days (1.40 ± 0.20 for G1 and 1.30 ± 0.25 for G2; *p* = 0.78) (Figure 3).

### 3.3 GAIS

Disregarding the product or plane applied, a positive GAIS score of 1.48 ± 0.599 was found at 60 days; however, it was significantly reduced to 1.15 ± 0.864 at 120 days assessment (mean difference = 0.33 ± 0.145; *p* = 0.031). Moreover, GAIS scores comparison between application planes at 60 days resulted in no significant differences (*p* = 0.895), with a subcutaneous application GAIS score of 1.5 ± 0.607 and supraperiostial application GAIS score of 1.45 ± 0.605.

**FIGURE 4:**
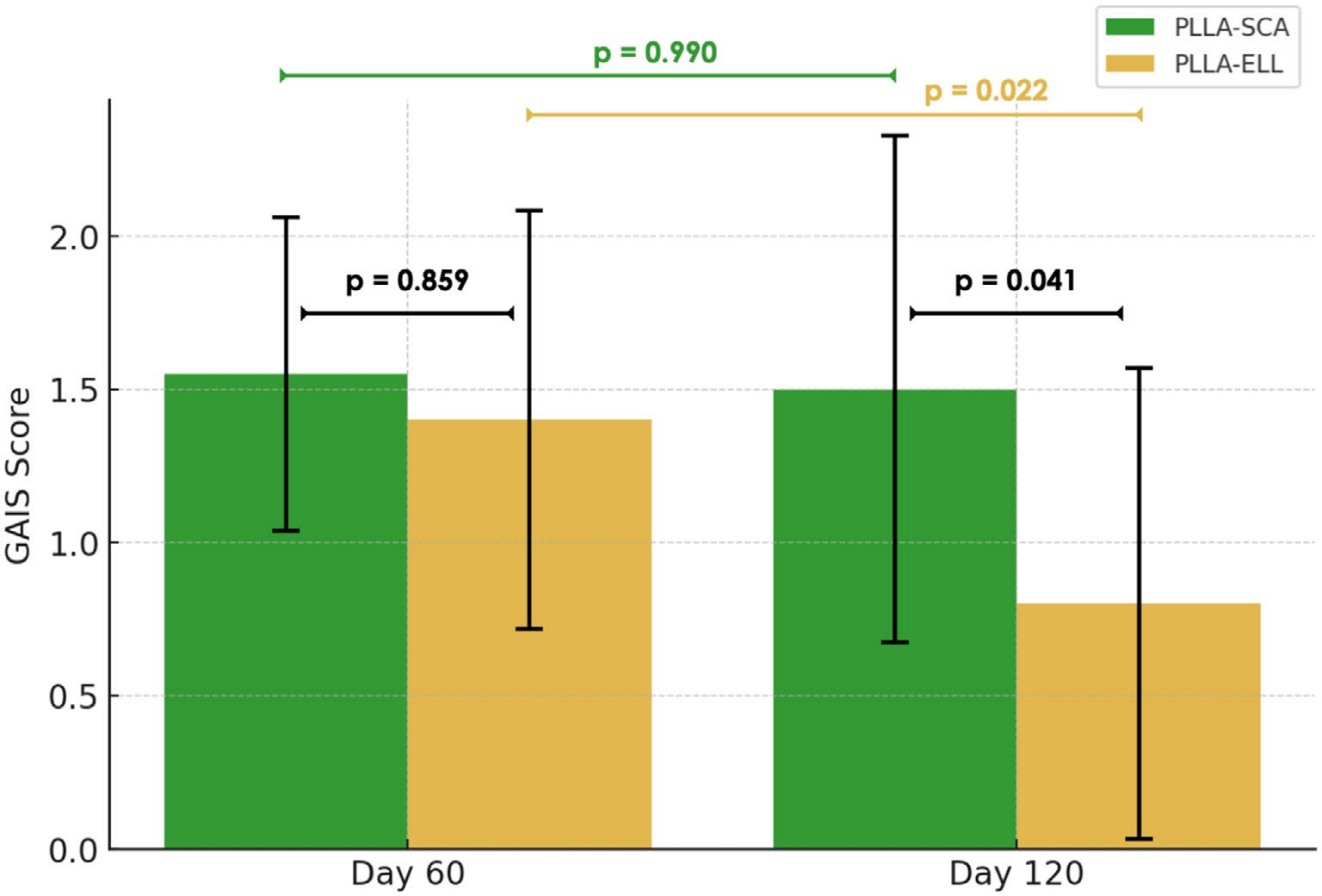
Bar graph showing the mean and standard deviation (SD) of GAIS scores at 60 and 120 days post‐treatment for PLLA‐SCA and PLLA‐ELL groups. P‐values indicate the significance of differences between time points according to the Wilcoxon Signed Rank test.

When comparing GAIS scores among the products used, at 60 days the mean GAIS values were similar between them (*p* = 0.859), with G1 presenting 1.55 ± 0.51 and G2 1.40 ± 0.68. However, at 120 days assessment G1 GAIS score was maintained with 1.50 ± 0.83 (*p* = 0.99 vs 60 days) while G2 GAIS score was significantly reduced to 0.80 ± 0.77 (*p* = 0.022 vs 60 days). Between groups, differences were found at 120 days with a significantly higher GAIS score for G1 compared to G2 (mean difference = 0.70 ± 0.25; *p* = 0.041) (Figure 4).

### 3.4 Correlation Between Dermal Thickness and GAIS

To explore the relationship between objective dermal thickness changes and patient‐perceived improvement, Spearman's correlation was performed between percentage change in dermal thickness (baseline to 60 and 120 days) and GAIS scores at corresponding time points.

The analysis revealed no significant correlation between dermal thickness change and GAIS at 60 days (*ρ* = −0.038, *p* = 0.819) or dermal thickness change and GAIS at 120 days (*ρ* = 0.016, *p* = 0.927), suggesting that increases in dermal thickness did not directly predict patient satisfaction. Furthermore, the correlation between GAIS 60d and GAIS 120d showed a weak but significant positive association (*ρ* = 0.325, *p* = 0.040), indicating that patients who reported a higher satisfaction at 60 days tended to maintain a positive perception at 120 days.

### 3.5 Adverse Events

Transient local reactions were observed in all participants. All patients presented minimal edema immediately after the procedure, which resolved spontaneously over the subsequent days without any intervention. Pain during the procedure was reported by four patients, and mild post‐treatment discomfort was reported by seven patients; however, no pharmacological or additional treatment was required in any case. These transient reactions occurred bilaterally, with no differences between hemifaces or injection planes.

One clinically relevant adverse event was observed, consisting of nodule formation following the first supraperiosteal application of PLLA‐ELL. The nodule was treated with intralesional triamcinolone acetonide (Triancil, Apsen Farmacêutica S.A, São Paulo, Brazil), with complete resolution after 15 days. No other persistent or clinically relevant adverse events were recorded.

## 4 Discussion

The present study aimed to compare the clinical outcomes of two commercially available PLLA‐based biostimulators, administered through subcutaneous and supraperiosteal injection techniques, in a split‐face design. Our findings revealed that, despite an overall trend of increased dermal thickness over time, no statistically significant differences were observed between treatment planes (at 60 days assessment) or products (at 120 days assessment). This suggests that the volumetric effects of PLLA, as assessed by high‐frequency ultrasound, may be independent of the specific formulation used. An initial improvement in facial aesthetics was observed in both groups, as reflected by positive GAIS scores at 60 days. Patients reported a noticeable enhancement in skin quality and overall appearance, consistent with the expected bioestimulatory effects of PLLA. However, this improvement was not sustained uniformly over time, as GAIS scores declined significantly at 120 days, particularly in the PLLA‐ELL group. In contrast, patient‐reported satisfaction remained relatively stable in the PLLA‐SCA group. These findings suggest that while both biostimulators provided early aesthetic benefits, differences in the persistence of patient‐perceived effects within the follow‐up period became evident over time.

In this context, it is important to emphasize that dose equivalence in the present study was defined based on the total amount of PLLA (mg) delivered per treated area, while each formulation was reconstituted strictly according to the manufacturer's recommendations. As a result, differences in reconstitution volume and particle dispersion were intentionally preserved, as they reflect real‐world clinical practice and may influence the spatial distribution and temporal profile of the bio‐stimulatory response, ultimately affecting patient‐perceived outcomes.

Several months after application, PLLA induces collagen production through a subclinical inflammatory response that promotes fibroplasia [10, 11]. While its effects develop progressively, histological studies have reported an early increase in collagen levels within the first few months. Goldberg et al., for instance, observed a significant 14.1% increase in type I collagen following PLLA‐SCA administration [12]. However, despite this quantitative histological evidence, their study did not assess patient‐reported outcomes or objective clinical changes. This highlights a crucial gap in the literature, as increased collagen deposition alone does not necessarily translate into visible aesthetic improvements or enhanced patient satisfaction.

The discrepancy between subjective (GAIS) and objective (dermal thickness) assessments may be attributed to improvements in skin quality that were not directly measured in this study. Additionally, dermal thickness was assessed in a single anatomical region (lateral zygomatic area), whereas GAIS reflects a global facial evaluation. While high‐frequency ultrasound provides valuable structural insights, it does not capture parameters such as hydration, elasticity, and transepidermal water loss, which are known to contribute significantly to patient satisfaction. A randomized, controlled, multicenter study previously demonstrated that PLLA‐SCA treatment led to significant improvements in hydration and skin elasticity after 12 months, along with reductions in pigmentation, erythema, and pore size [6]. These enhancements may explain why some patients maintained a positive perception of their results despite the absence of measurable volumetric changes. Supporting this notion, correlation analysis in our study revealed no significant relationship between percentage change in dermal thickness and GAIS scores at either 60 or 120 days. This suggests that increases in dermal thickness alone do not necessarily translate into greater patient satisfaction. Instead, factors such as skin texture, elasticity, and hydration, none of which were assessed in this study, may exert a more substantial influence on subjective aesthetic perception. Therefore, even if confirmed in larger samples, small changes in dermal thickness may not necessarily represent clinically meaningful improvement from the patient's perspective.

Importantly, the divergence between objective and subjective evaluations is not uncommon in aesthetic medicine. While clinicians often rely on quantifiable changes to assess treatment efficacy, a recent prospective study emphasized that the true clinical relevance of an intervention ultimately depends on patient satisfaction [5]. Even when statistically significant improvements in dermal parameters are observed, they may hold little value if they do not align with the patient's expectations and perceived aesthetic enhancement [13, 14]. Supporting this concept, Spearman's correlation in our study revealed a weak but significant positive association between GAIS scores at 60 and 120 days, indicating that patients who initially perceived an improvement were more likely to maintain a positive perception over time. This finding underscores the influence of psychological and perceptual factors on patient satisfaction, reinforcing that clinical relevance should not be determined solely by objective volumetric changes but also by subjective experiences and expectations.

A progressive decline in GAIS scores at 120 days may be partially explained by the well‐documented phenomenon of hedonic adaptation, in which individuals gradually become accustomed to aesthetic improvements, leading to a reduced perception of their initial impact [15]. However, beyond this general psychological effect, the differential decline in GAIS scores between products suggests that formulation‐specific physicochemical characteristics represent the predominant mechanism underlying the faster reduction in perceived effect observed with PLLA‐ELL. In this context, the differences in GAIS scores between PLLA‐SCA and PLLA‐ELL at 120 days may be attributed to their distinct physicochemical properties, which influence their degradation kinetics and bioavailability. Sedush et al. demonstrated that PLLA‐SCA has a lower molecular weight (78 kDa) and crystallinity (64%) compared to PLLA‐ELL, which exhibits higher molecular weight (114 kDa) and crystallinity (72%) [16]. These characteristics suggest that PLLA‐SCA undergoes a more gradual and sustained degradation, potentially contributing to differences in the temporal evolution of the bioestimulatory response. Conversely, PLLA‐ELL, with its higher crystallinity and smaller, more porous particles, may degrade more heterogeneously, resulting in an initially more intense response followed by a faster decline in perceived effect. This more pronounced early inflammatory stimulus, plausibly associated with higher crystallinity and heterogeneous degradation, may also contribute to localized tissue reactions in susceptible contexts, such as the isolated nodule observed following supraperiosteal PLLA‐ELL application in the present study. These findings highlight that not all PLLA‐based biostimulators behave identically in clinical practice, reinforcing the importance of considering their molecular and structural properties when selecting a product for long‐term facial rejuvenation.

Another important aspect to consider is the lack of significant differences between the supraperiosteal and subcutaneous injection planes at 60 days assessment. This finding is consistent with the results reported by Mazzuco et al., who also compared PLLA injections at different depths and found no statistically significant differences between them. Although their study suggested a slight trend toward greater dermal and subcutaneous thickness on the subcutaneous side, these differences did not reach statistical significance, likely due to the small sample size (*n* = 6). In contrast, our study included a larger cohort (*n* = 20), providing a more robust statistical framework to assess the impact of injection depth on dermal remodeling. These findings suggest that the efficacy of PLLA in stimulating collagen deposition, on a short term basis, may be largely independent of the injection plane, reinforcing its versatility in clinical practice [17].

Despite its valuable contributions, this study has some limitations that should be acknowledged. First, although the sample size (*n* = 20) was adequate for the within‐subject, split‐face comparison of injection planes, it may have limited the detection of smaller differences in the between‐product (parallel‐group) analysis, potentially affecting the generalizability of inter‐product comparisons. Second, the follow‐up period of 120 days may not fully capture the long‐term effects of PLLA, as collagen biostimulation can continue for several months after treatment; moreover, although instrument‐related effects were balanced by the split‐face crossover design, the use of different delivery devices (needle vs. cannula) may still represent a residual confounding factor for collagen stimulation and should be further addressed in future studies. Finally, the present investigation focused on dermal thickness and overall patient‐perceived aesthetic improvement assessed by GAIS. While this approach was intentional and aligned with the study objective of contrasting global perception with objective ultrasound findings, other skin‐related parameters (e.g., elasticity, hydration) and multidimensional patient‐reported outcomes were not evaluated. Future studies incorporating additional objective measures and validated domain‐specific PROMs may provide a more comprehensive characterization of treatment effects.

## 5 Conclusion

Our findings suggest that patient‐perceived improvements after PLLA treatments may not be solely dictated by measurable volumetric changes, but rather by a combination of factors, including skin quality enhancements and individual perception dynamics. This underscores the importance of integrating both objective and subjective assessments when evaluating the efficacy of biostimulatory treatments.

Beyond application techniques, our results also highlight the critical role of physicochemical properties in shaping the longevity of perceived aesthetic benefits. The sustained satisfaction observed with PLLA‐SCA compared to PLLA‐ELL suggests that formulation characteristics may directly impact patient‐perceived outcomes within the observed follow‐up. Therefore, the selection of a PLLA formulation should extend beyond bioactivity alone, considering its long‐term influence on patient satisfaction.

## Author Contributions

The authors V.R.M.M.‐L. and M.G. contributed to developing the concept for the work; acquiring, analyzing, and interpreting the data; drafting the article; and revising it critically for important intellectual content. They also granted final approval to the version to be published and agreed to be accountable for all aspects of the work in ensuring that questions related to the accuracy or integrity of any part of the work were appropriately investigated and resolved. The authors D.D., F.B., A.C.N.C., R.V., A.D.T., P.R., G.G, P.P, and V.R. contributed to the acquisition and interpretation of data and critically revised the article for important intellectual content.

## Funding

This investigation received no funding. The products utilized in this study were donated by the authors for the purposes of this study.

## Ethics Statement

This clinical study was approved by the Research Ethics Committee of CENTRO UNIVERSITÁRIO DE JAGUARIÚNA—UNIFAJ (Approval number: 6.240.623; CAAE: 68933023.1.0000.0191) on February 20, 2024. All procedures were conducted in accordance with the Declaration of Helsinki and relevant national regulations. Written informed consent was obtained from all participants prior to their inclusion in the study. All participants were informed about the potential risks and benefits of the procedure and were guaranteed confidentiality throughout the data collection and publication process.

## Conflicts of Interest

D.D., R.V., A.D.T., V.R., V.R.M.M.‐L., and M.G. are speakers for Galderma. However, Galderma did not provide financial support for this study. The remaining authors declare no conflicts of interest related to this research.

## Data Availability

The data supporting the findings of this study are fully reported within the article.
